# Towards Optimizing Neural Network-Based Quantification for NMR Metabolomics

**DOI:** 10.3390/metabo15040249

**Published:** 2025-04-04

**Authors:** Hayden Johnson, Aaryani Tipirneni-Sajja

**Affiliations:** 1 Department of Biomedical Engineering, The University of Memphis, Memphis, TN 38152, USA; 2 Department of Biomedical Engineering, University of Houston, Houston, TX 77004, USA

**Keywords:** NMR spectroscopy, convolutional neural network, multi-layered perceptron, transformer, low-field NMR

## Abstract

**Background:** Quantification of metabolites from nuclear magnetic resonance (NMR) spectra in an accurate, high-throughput manner requires effective data processing tools. Neural networks are relatively underexplored in quantitative NMR metabolomics despite impressive speed and throughput compared to more conventional peak-fitting metabolomics software. **Methods:** This work investigates practices for dataset and model development in the task of metabolite quantification directly from simulated NMR spectra for three neural network models: the multi-layered perceptron, the convolutional neural network, and the transformer. Model architectures, training parameters, and training datasets are optimized before comparing each model on simulated 400-MHz ^1^H-NMR spectra of complex mixtures with 8, 44, or 86 metabolites to quantify in spectra ranging from simple to highly complex and overlapping peaks. The optimized models were further validated on spectra at 100- and 800-MHz. **Results:** The transformer was the most effective network for NMR metabolite quantification, especially as the number of metabolites per spectra increased or target concentrations were low or had a large dynamic range. Further, the transformer was able to accurately quantify metabolites in simulated spectra from 100-MHz up to 800-MHz. **Conclusions:** The methods developed in this work reveal that transformers have the potential to accurately perform fully automated metabolite quantification in real-time and, with further development with experimental data, could be the basis for automated quantitative NMR metabolomics software.

## 1. Introduction

Neural networks (NNs) have enabled unparalleled speed and automation in many fields like natural language processing and computer vision and have achieved impressive feats in biology, such as AlphaFold solving challenges in protein folding prediction [1]; however, these techniques have not gained much traction in the task of metabolite identification and quantification directly from nuclear magnetic resonance (NMR) spectra. NNs are especially promising for the field of NMR metabolomics, given the complexity of NMR spectra and the time and expertise required to process the spectrum into a list of quantified metabolites. NNs have seen scattered use for analyte quantification directly from spectra in scientific publications over the past three decades [2,3,4], and a recent resurgence [5,6,7] has been driven by improvements and innovation in artificial intelligence techniques, computing power, and NMR spectroscopy. This study aims to improve the utility of NNs for NMR metabolomics profiling in complex samples with a high dynamic range of concentrations by investigating methods in dataset development, NN architecture, and model training.

In NMR-based metabolomics research, metabolite identification and quantification are often tedious tasks involving several manual steps. A fully manual process involving resonance deconvolution can easily take up to an hour for complex samples and is limited by human errors. Even if assisted by the popular lineshape fitting software, Chenomx [8] (https://www.chenomx.com, (accessed on 10 January 2025)), it can take up to 40 min to process a complex sample, and the semi-automated version of Chenomx can speed this up to ~1 min per spectrum at the cost of reduced accuracy [9]. Fully automated software like MagMet (https://www.magmet.ca, accessed on 4 March 2024) can quantify metabolites in serum or plasma in ~2–3 min [9]. However, none of these approaches used in NMR metabolomics research can match the speed and scalability of a popular class of machine learning algorithms, NNs, which have been used to quantify the lipid profile in 5000 spectra in under 1 s [7]. NNs are fully automated, rapid, and scalable technologies that can be applied for processing NMR data. NNs have already been applied to a variety of NMR processing tasks, including denoising spectra [10], reconstructing non-uniformly sampled data [11,12], resonance characterization [13,14], and analyte quantification [5,6,7]. In the task of analyte quantification, networks such as multi-layered perceptrons (MLPs) [5,7], convolutional neural networks (CNNs) [5], and convolutional recurrent neural networks (CRNNs) [6] have been explored in a few studies to date; however, the application of NNs in metabolite profiling has been limited compared to software using statistical lineshape fitting approaches for metabolite profiling [9,15,16,17,18,19,20,21,22]. In this research, we intensively investigate methods applying NNs to metabolite profiling to determine good practices for accurate quantification. We apply MLP and CNN models for metabolite quantification in simulated 1D ^1^H-NMR spectra of complex mixtures. We also implement a NN architecture, the transformer, which has proven exceptionally proficient at a large variety of complex tasks like natural language processing [23] and vision tasks [24], however, it has not yet been investigated in the context of analyte quantification for spectroscopic data.

For any NN, there are many considerations that affect the model’s performance. The dataset used to train a model is of major importance. In this study, we examine how varying concentration distributions used for model training affect the accuracy of NNs and further explore dataset modifications like varying the number of metabolites per spectra, log transforming spectral intensities, and more. As model architecture and training parameters also significantly drive model performance, we employ Bayesian hyperparameter optimization to improve the architectures as well as learning parameters for the MLP, CNN, and transformer models.

This study explores whether the MLP, CNN, or transformer is best suited for analyte quantification in 1D NMR spectra and validates some good practices in dataset development and parameter selection for NNs as applied to NMR-based metabolomics. The models developed throughout this study on simulated 400-MHz data are further validated in spectra of varying complexity (8, 44, and 86 metabolites—representing a range from simple spectra to the upper end of quantified metabolites seen in ^1^H-NMR metabolomics studies) and on simulated 100-MHz spectra and 800-MHz spectra, which are relevant with the rise of benchtop instruments [25,26,27] and availability of more high-field NMR spectrometers, respectively. The methods developed in this study are presented as a general workflow for developing NNs for NMR metabolite profiling, and suggestions are provided in the discussion for further improving and adapting this workflow for application on experimental NMR spectra. The results of this study are promising and show NNs can be extremely rapid and accurate for the quantification of analytes in highly complex NMR spectra, but there are limitations provided in the discussion that need to be considered and overcome if NNs are to become the tool of choice for quantitative NMR metabolomics practitioners.

## 2. Methods

**Figure 1:**
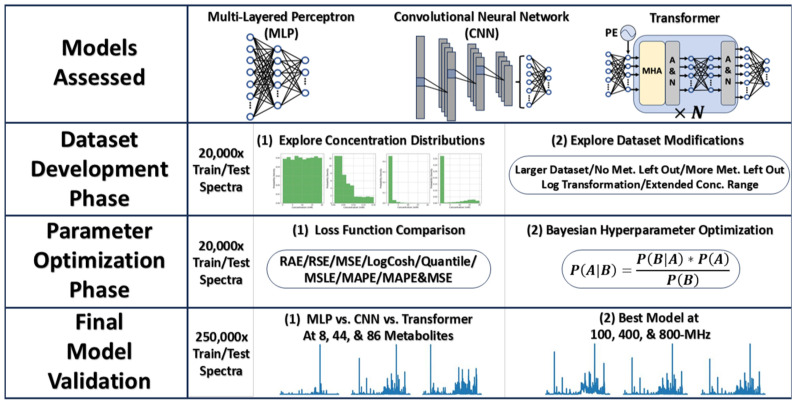
Workflow to study neural network performance in the task of analyte quantification from simulated NMR spectra of mixtures of 44 metabolites. Three types of networks (MLP, CNN, and transformer) were considered during dataset development and parameter optimization phase before a final comparison of the three models, and the best model was further validated on NMR spectra simulated at three different magnetic field strengths. Abbreviations: Met. = Metabolites, Val. = Validation, MLP = multi-layered perceptron, CNN = convolutional neural network, RAE = relative absolute error, RSE = relative squared error, MSE = mean squared error, LogCosh = logarithm of hyperbolic cosine error, MSLE = mean squared logarithmic error, MAPE = mean absolute percent error, PE = positional encoding, MHA = multi-headed attention, and A&N = add and norm.

To explore best practices in developing accurate neural networks for quantification of analytes directly from 1D ^1^H-NMR spectra, we developed networks using three promising architectures, the MLP, CNN, and transformer and examined model performance while varying dataset concentration distributions, dataset modifications, and loss functions, respectively, before optimizing hyperparameters through Bayesian optimization. The best model was further validated on simulated spectra at low- and high-field magnetic field strengths. An overview of this entire process is given in Figure 1.

### 2.1. Data Generation Methods

Simulated 400-MHz ^1^H-NMR spectra of complex metabolite mixtures were used to train neural networks for metabolite profiling. Mixture spectra were generated using simulated metabolite reference spectra downloaded from the human metabolome database (HMDB) [https://hmdb.ca, accessed 1 February 2024] [28]. Simulated spectra in the HMDB were calculated by predicting chemical shifts using HOSE-code (Hierarchically Ordered Spherical Environment code) and machine learning methods, estimating coupling with empirical rules, followed by spin matrix calculations [28]. Signals for 87 metabolites commonly encountered in biological aqueous tissue extracts were downloaded (see Table S1 in the Supplement). Representative spectra for these metabolites, along with a synthetic mixture spectrum containing all these metabolites, can be found in the supplement as Figure S1 (with alpha-D-glucose and beta-D-glucose anomers combined into a single spectrum for a total of 86 metabolites). The testing spectra generated in this study are based on aqueous metabolite mixtures seen in animal tissues, and several measures were taken to promote realistic spectra, including maintaining concentration magnitudes and distributions seen in real metabolomics samples, maintaining a signal-to-noise ratio (SNR) similar to those measured in real spectra, incorporating interfering signals at random, incorporating experimental variations (in line-broadening, noise, chemical shifts, and baseline), and combining simulated spectra for glucose anomers into one unified glucose spectrum. Data processing in this study was achieved with Python [29] (version 3.11.5) using the numpy [30] library (version 1.24.3) for most operations on matrices and the nmrglue [31] library (version 0.10) for NMR processing operations (file reading, shifting peaks, and Fourier transformations). Seaborn [32] (version 0.12.2) was used to generate point plots (plots showing mean and 95% confidence interval). All computations in this study were executed using a Quadro RTX 6000 (Nvidia, Santa Clara, CA, USA) and two Xeon Silver 4216s processors (Intel, Santa Clara, CA, USA).

Spectra downloaded from the HMDB exhibit no noise and have resonance intensities that are consistent with proton numbers contributing to each resonance; thus, each simulated spectrum was assumed to be 1 mM as downloaded from the HMDB, and any scaling produced analyte concentrations with the same magnitude as the scalar. To mimic 3-(Trimethylsilyl)propionic-2,2,3,3-d4 (TSP-d4) salt, a singlet was added at 0.0 ppm and scaled to a concentration of 0.3 mM (for 9 protons) in all spectra generated throughout the study.

**Figure 2:**
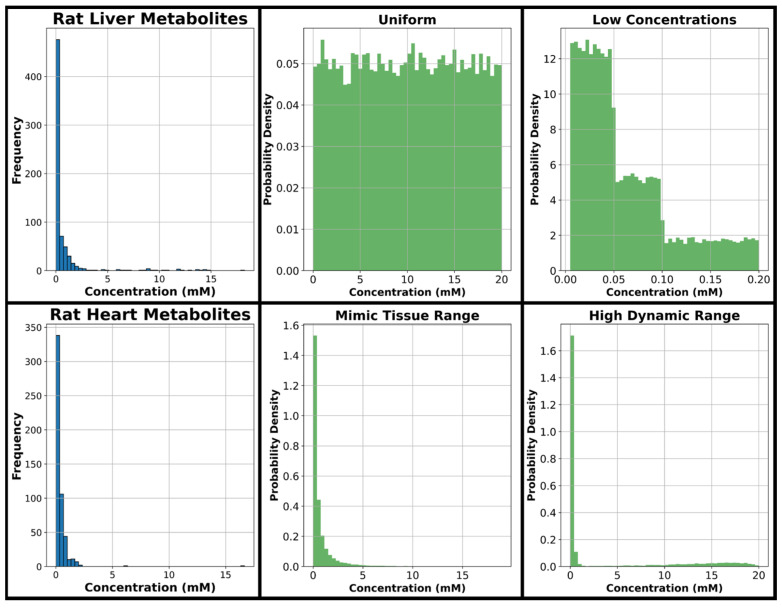
Left two blue histograms display the probability density of concentrations from a murine metabolomics study [33] and the remaining four green histograms show the primary concentration distributions used in generating simulated complex mixtures of metabolites.

The range of concentrations considered in data generation was 0.005–20 mM based on our previous experimental work in rat liver and heart [33] tissue extracts measured using a 400-MHz NMR spectrometer. Histograms displaying metabolite concentration frequencies for murine liver and heart tissue samples in our previous study can be seen in Figure 2, along with four histograms displaying four concentration distributions utilized in this study. The concentration distribution the model sees in training is expected to have a major effect on model performance; therefore, for model training, a uniform distribution is considered covering the entire range of metabolite concentrations evaluated in this study, and several further concentration distributions are considered emphasizing the lower concentrations common in metabolomics spectra which are expected to be more difficult for quantification given their relatively small size in the overall spectra. One distribution is considered with exclusively low concentrations, while two different distributions with a large dynamic range emphasizing low concentrations are evaluated given they are expected to more closely resemble NMR metabolomics spectra of tissue extracts.

The four green histograms in Figure 2 are the major concentration distributions used in generating training, testing, and validation data throughout this manuscript. The distribution referred to as “uniform” in this manuscript uniformly encompasses the full range from 0.005 to 20 mM, and this uniform approach has been used in several previous NN analyte profiling studies [5,7]. The distribution referred to as “low concentration” in this manuscript is an evenly split combination of three uniform distributions that each begin at 0.005 mM and extend to 0.05, 0.1, and 0.2 mM, respectively. Two distributions were created with concentration distributions similar to those seen in tissues where several metabolites have relatively high concentrations and most have much lower concentrations, an approach similar to the one taken by Wang et al. [6]. The first is referred to as “mimic tissue range” and is a combination of two log-normal distributions, and the second is referred to as “high dynamic range” and is a combination of two gamma distributions and one uniform distribution. The code used to generate each of these four distributions is found in this project’s GitHub repository (https://github.com/tpirneni/DL-NMR/blob/main/DefineDistributions.ipynb, accessed 10 January 2025). A fifth concentration distribution was developed by using all four of the aforementioned distributions, and this is referred to as the “combined distribution” (i.e., for the combined distribution, 25% of samples are generated using the uniform distribution, 25% are generated using the low concentration distribution, 25% come from the mimic tissue range distribution, and 25% come from the high dynamic range distribution). All five concentration distributions are used to produce training and validation datasets in this study. The uniform, low concentration, and mimic tissue range distributions are used to generate testing datasets of 10 spectra each throughout this study for comparing model accuracies.

For determining realistic SNR magnitude, two aqueous reference standards were weighed targeting concentrations near 1 mM, prepared for NMR in a solution of deuterium oxide buffered to a pH of ~7.4, and scanned on a 400-MHz JEOL NMR spectrometer using a 1D-NOESY pulse sequence with 32 averages, water pre-saturation, a pulse angle of 90°, and a relaxation delay of 15 s. Receiver gain was set at a level to accommodate metabolite concentrations of at least 20 mM for maintaining a realistic SNR dynamic range (determined using autogain in preliminary experiments). Choline (as choline chloride) was purchased from Acros Organics (Geel, Belgium) and was prepared at a concentration of 0.92 mM, and creatine (as creatine monohydrate) was purchased from Acros Organics and was prepared at 1.0 mM. SNR was determined for these experimentally acquired scans by dividing the height of the tallest peak by the standard deviation of the noise. Simulated spectra of choline (scaled by 0.92) and creatine (unscaled) were subjected to the addition of various levels of normally distributed noise until similar SNR values were achieved, and the comparison between experimental and simulated spectra along with computed SNRs can be seen in Supplementary Figure S2.

A data augmentation workflow was implemented for all spectra generated to produce greater variation in the datasets and imitate potential experimentally encountered signal variations, including adding normally distributed noise (magnitude ranging from 30 to 115% of the noise level determined previously using experimental choline and creatine spectra), varying line-broadening (using exponential apodization values ranging uniformly from 0 to 1), slightly shifting entire metabolite signals along the chemical shift axis (ranging uniformly from 0 to 3.4 ppb left or right per analyte), slightly shifting the baseline up or down (up to ~5.6% of the reference TSP-d4 peak height), and the addition of up to three singlets at random chemical shifts scaled using a gamma distribution promoting mostly concentrations under 5 mM with a mean of 0.85 mM (using the acetic acid signal for a generic singlet, see interference concentration distribution in https://github.com/tpirneni/DL-NMR/blob/main/DefineDistributions.ipynb, accessed 10 January 2025). Many of these modifications can be seen in Supplementary Figures S3–S5, which show zoomed-in 100-, 400-, and 800-MHz spectra, respectively, of overlapping 1 mM NADP and nicotinic acid mononucleotide resonances expressing many of the augmentations applied above independently (max peak shift, max noise added, min noise added, max line-broadening, and max base-shift). The following three figures in the Supplement (Figures S6–S8) display the full spectra of 44 metabolites with the same modifications as in the preceding figures. As D-glucose in aqueous solutions typically reaches an equilibrium of ~36% alpha-D-glucose and ~64% beta-D-glucose [34], we scaled the simulated spectra of these anomers by 0.36 and 0.64, respectively, before combining them into a single simulated glucose spectrum which was used in generating training, testing, and validation data. For all metabolites, only the region from −0.32 through 10.21 ppm containing 46,000 data points is considered for data generation.

A total of 44 metabolites are considered analytes in this manuscript (see 45 mentioned in Supplementary Table S1, with two glucose anomers combined). All spectra are scaled to intensity values between zero and one by dividing all training, testing, and validation spectra by the maximum intensity value determined per dataset generated. A brief demonstration of the general data generation workflow with several spectra displayed can be found in the README.md file of this project’s GitHub page (https://github.com/tpirneni/DL-NMR, accessed 10 January 2025). All datasets are split 80:20 for training and validation, respectively.

### 2.2. Neural Network Development

All models in this manuscript were developed and trained using Pytorch [35] (version 2.2.1) [https://pytorch.org/ accessed 1 February 2024]. Preliminary investigations of MLP, CNN, and transformer models for analyte quantification led to the initial set of model architectures and training parameters used throughout the dataset development and loss function phases. The MLP was instantiated with 46,000 input nodes (corresponding to all data points in synthetic spectra), a 200-node hidden layer, and 44 output nodes and was trained using a learning rate of 0.001 and batch size of 169. The CNN model was instantiated with four consecutive blocks of a convolutional layer of 42 kernels (stride of 2, size of 6) followed by ReLU activation and max pooling (stride of 2, size of 2), followed by 2 feedforward layers of 200 and 44 nodes, respectively. The CNN used a learning rate of 5.3 × 10^−5^ and a batch size of 64.

For the transformer model, a modified version of the transformer architecture described by Vaswani et al. [36] was instantiated as an encoder-only transformer followed by a single linear layer. The 46,000 data points of input spectra were split into 46 bins with 1000 data points each, with model inputs considered sequences with length 46 and 1000 features each. The first layer of the transformer was a linear layer of 512 nodes for computing embeddings, followed by positional encodings as applied by Vaswani et al. [36]. Positionally encoded matrices are passed into a 1-layer transformer encoder with 1 attention head, a feedforward dimension of 2048 nodes, and with no dropout applied. Encoder outputs are flattened and passed into a final feedforward layer with 44 nodes. A batch size of 76 and a learning rate of 1.5 × 10^−4^ are used for the transformer. A code demonstration of the development of a simple transformer encoder, along with application to some synthetic spectra, can be found in the README.md file of this project’s GitHub page (https://github.com/tpirneni/DL-NMR, accessed 10 January 2025).

Throughout the dataset development and loss function phases, the AdamW optimizer is used for training all models with a regularization constant of 0.01. Relative absolute error (RAE) was the loss function used in the dataset development phase. All models were trained until 50 epochs passed without achieving a new best validation loss value or until 5000, 500, or 1000 epochs passed for the MLP, CNN, and transformer, respectively.

### 2.3. Dataset Development Phase

As the performance of any neural network depends heavily on the training dataset, we performed an investigation of how varying the concentration distribution and otherwise modifying the training dataset might affect model performances. Concentration distributions were explored first by training the MLP, CNN, and transformer models on datasets using the uniform, low concentration, mimic tissue range, high dynamic range, and combined distribution. Five training and validation datasets of 20,000 spectra were generated by scaling the 44 analytes using scalars pulled from one of the five above-mentioned distributions (one dataset per distribution: uniform, low concentration, mimic tissue range, high dynamic range, and combined distribution). For each dataset, 10,000 spectra included all 44 analytes every time, while 10,000 spectra had a 50% chance to leave out any analyte. Ten testing spectra were generated using the uniform, low concentration, and mimic tissue range distributions, and all 44 metabolites are included to facilitate computation of mean absolute percent error (MAPE), which is used as the metric for comparing model performances on the testing spectra, with mean MAPE used to determine the best performing models.

After comparing concentration distributions, the best distribution was used to compare several dataset modifications for their effects on accuracy for the MLP, CNN, and transformer models. The first modification applied to the training dataset was the log-transformation which was applied to all spectra as a means of decreasing the dynamic range of intensities between the largest and smallest peaks. The transformation is described by the equation, $L=\log_{10}\left(1+S\times 1000\right)$, where ‘*L*’ represents the log-transformed spectra intensity and ‘*S*’ is intensity in the original spectrum; an example of this transformation is shown in Supplementary Figure S10.

The second dataset modification involved simply increasing the dataset size from 20,000 to 50,000. The third dataset modification was to not leave any metabolites out of the spectra (as opposed to 10,000 spectra having a 50% chance to leave out any metabolite). The fourth dataset modification was to leave out even more metabolites by having 1/3 of spectra, including all 44 metabolites, 1/3 of spectra having a 50% chance to leave out any metabolite, and 1/3 of spectra having a 75% chance to leave out any metabolite. The fifth dataset modification examined was extending the upper limit of the concentration range from 20 mM to 24 mM (i.e., generating data within the range 0.005 to 24 mM rather than 0.005 to 20 mM). MAPE determined for each testing example in the uniform, low concentration, and mimic tissue range test spectra were normalized by the MAPE determined by the respective model prior to dataset modification (i.e., MAPE determined on the uniform test spectra using the MLP trained on log-transformed is divided by the test MAPE determined by the MLP trained on the unmodified dataset, and so on for all models and testing examples). In this way, modifications with a mean normalized MAPE lower than 1.0 improved performance, and modifications with a mean normalized MAPE higher than 1.0 worsened performance.

### 2.4. Parameter Optimization Phase

The best concentration distribution, along with the beneficial dataset modifications determined in the above-mentioned experiments, was used to generate 20,000 spectra for training the MLP, CNN, and transformer models using several different loss functions, including RAE, relative squared error (RSE), mean squared error (MSE), quantile loss (with q = 0.1, 0.5, and 0.9), logarithm of hyperbolic cosine (LogCosh) loss, mean squared logarithmic error (MSLE), MAPE, and a 50:50 combination of MSE and MAPE (MSE&MAPE). For the MAPE and MSE&MAPE, no metabolites were left out of any dataset, as concentrations of zero would not permit MAPE calculation. MAPE determined by each model on the 10 uniform, low concentration, and mimic tissue range test spectra was used to compare loss function performances.

The improved dataset of 20,000 spectra and loss function determined in the above-mentioned experiments were utilized next in the hyperparameter optimization phase. Optuna [37] (version 3.6.1) [https://optuna.org, accessed 1 February 2024] was used to tune hyperparameters using the TPESampler algorithm (tree-structured Parzen estimator Bayesian hyperparameter optimization available in Optuna) to determine model-specific parameters for the MLP, CNN, and transformer, and the parameter space searched for each model listed in Table 1. Each model was subjected to 100 trials (where a set of hyperparameters is used to train a model) using the same number of epochs as in previous experiments. Different pruning, or early stopping criteria, were useful when training different models. For the MLP, the median pruner in Optuna, which stops a trial if the loss at any step is worse than the median of results of prior trials at that timestep, was used as an early stop criterion using validation loss. For the CNN, the threshold pruner in Optuna was used to develop early stop criteria using validation loss (if loss is greater than 5000 after 15 epochs or greater than 1000 epochs after 100 epochs, then that trial is pruned). For the transformer, the threshold pruner was used to stop trials early if 100 epochs pass and loss is greater than 1000. Despite using the above-described validation loss for pruning trials, the objective function was defined to minimize MAPE on a further validation dataset of 5000 spectra generated without leaving out metabolites for both the MLP and transformer. Quantile loss was minimized on the same validation set for the CNN, since attempts using MAPE were resulting in overfitting for the CNN. In hyperparameter optimization and in the remainder of this study, gradients were accumulated over four small batches per epoch to allow for larger batch sizes given computational resource limitations, and batch size is reported as effective batch size to reflect this.

The best parameter set determined through hyperparameter optimization was then used to train an MLP, CNN, and transformer, each using a large dataset of 250,000 spectra. Each model was trained and validated on spectra containing 8, 44, or 86 metabolites (as noted in Supplementary Table S1). To conclude which model had the superior performance in this study, MAPE was determined on 10 uniform, low concentration, and mimic tissue range test spectra.

### 2.5. Model Testing for Low- and High-Field Simulated Spectra

The best dataset distribution and modifications from prior experiments were used to generate datasets of 250,000 spectra at 100-MHz and 800-MHz using simulated spectra downloaded from the HMDB of the same 45 metabolites (43 analytes and 2 glucose anomers that combine for 44th analyte). The only difference in the data generation processes was to increase noise by 8 times in the 100-MHz spectra and decrease noise by 2.8 times in the 800-MHz spectra to simulate different potential SNRs at varied field strengths (SNR scaled by the power of 3/2) [38,39]. In addition to changes to the signal-to-noise ratio of spectra, changing the magnetic field strength also affects the spectral resolution of NMR data. As field strengths increase, signals increase in resolution, and peaks are better separated along the chemical shift axis [40]. Conversely, as field strengths decrease, there is lower spectral resolution, which is especially a problem for complex NMR metabolomics samples as signals become more and more overlapped and, thus, more difficult to identify and quantify [41]. Only the best model determined in hyperparameter optimization was trained for quantification of analytes at 100- and 800-MHz, and accuracy was determined on uniform, low concentration, and mimic tissue range testing spectra. MAPE from these models was compared to the corresponding model trained for quantification at 400-MHz.

## 3. Results

### 3.1. Dataset Development Phase

**Figure 3:**
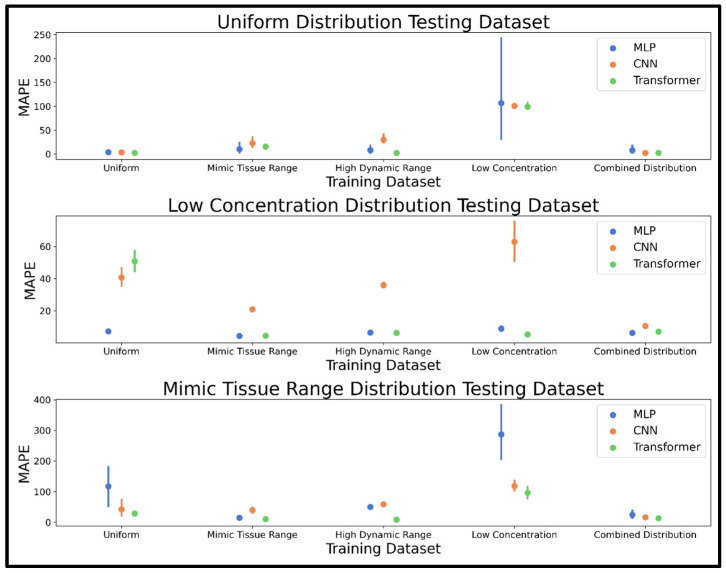
Pointplots displaying the mean and 95% confidence interval of the MAPE determined for the MLP, CNN, and transformer models trained using five different training datasets of 20,000 spectra with varied concentration distributions, including uniformly distributed, a tissue-mimicking range, a high dynamic range, a low concentration range, and a combined distribution. MAPE was determined on 10 test spectra generated using the uniform, low concentration, and mimic tissue range distributions as shown in the top, middle, and bottom panels, respectively. Abbreviations: MAPE = mean absolute percent error; MLP = multi-layered perceptron; CNN = convolutional neural network.

The training/validation dataset concentration distribution was the first parameter varied to study its effect on NN model accuracy. Accuracy metrics reported as mean MAPE and 95% confidence interval are shown in Figure 3, while Table S2 in the Supplement provides mean MAPE values. Several models achieved high accuracy with a mean MAPE of under 5% on the uniformly distributed concentration test example, including the transformer (trained on the uniform, high dynamic range, and combined distributions), the CNN (trained on the combined and uniform distributions), and the MLP (trained on the uniform distribution). For the low concentration test spectra, only the MLP and transformer trained on the mimic tissue range dataset achieved under 5% mean MAPE, followed closely by the low concentration distribution trained transformer at 5.2% mean MAPE. For the mimic tissue range distribution test spectra, the transformer was the most accurate model with training on the high dynamic range, mimic tissue range, and combined distributions, achieving mean MAPEs of 8.3, 10.1, and 12.9%, respectively. The combined distribution dataset was the most consistent in achieving high accuracy for all models and testing datasets, so this dataset was selected for the next phase of dataset development. All three neural network architectures were extremely fast, capable of processing 5000 spectra in 1, 2, and 12 ms, respectively, for the MLP, CNN, and transformer models.

**Figure 4:**
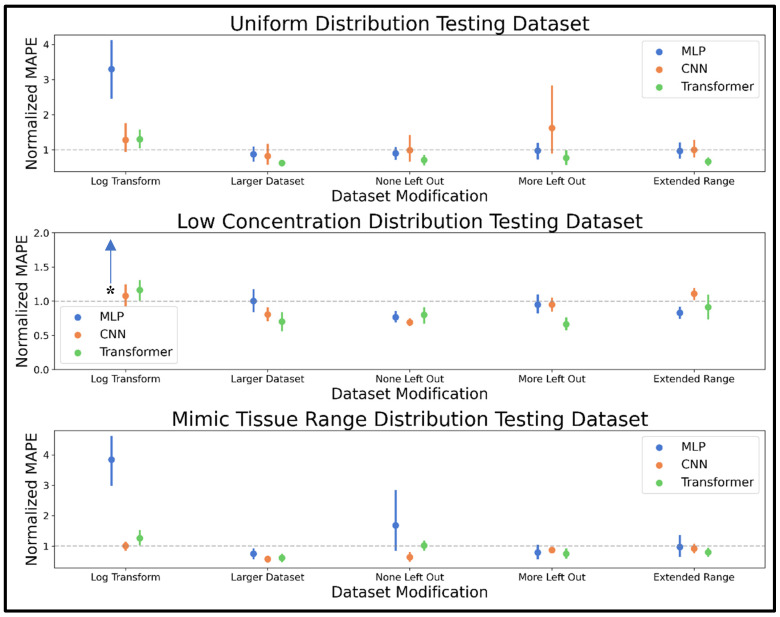
Pointplots displaying the mean and 95% confidence interval of the normalized MAPE determined for the MLP, CNN, and transformer models trained using five training datasets of 20,000 spectra with different modifications with respect to the combined distribution dataset, including log transformation (Log Transform), increasing to 50,000 spectra (Larger Dataset), leaving no metabolites out of any spectra (None Left Out), leaving more metabolites out of some spectra (More Left Out), and extending the upper limit of the concentration range (Extended Range). MAPE is normalized by the MAPE determined on the combined distribution dataset and was determined on 10 test spectra generated using the uniform, low concentration, and mimic tissue range distributions as shown in the top, middle, and bottom panels respectively. The grey dotted line at y = 1 in each panel denotes even accuracy for the current model compared to the model trained without dataset modification. The asterisk (*) denotes that the data point denoting MLP is at a high normalized MAPE value, which was left out to keep the y-axis range visually reasonable (normalized MAPE of ~10.1). Abbreviations: MAPE = mean absolute percent error; MLP = multi-layered perceptron; CNN = convolutional neural network.

The combined distribution dataset was modified using five approaches, including log transformation of input spectra, increasing the dataset size by 150%, not leaving any metabolites out of any spectra, leaving more metabolites out of some spectra, and extending the range of concentrations seen in training. Mean MAPE results on the 10 uniform, low concentration, and mimic tissue range distribution test spectra were normalized by the same metric determined prior to dataset modification, and these normalized MAPE results are shown in Figure 4, while the actual values are displayed in Supplementary Table S3. The log transformation resulted in mean normalized MAPE values greater than 1.0 for all three models on all three datasets. Increasing the dataset size improved the quantitative performance of all models, with the largest effect for the transformer and the smallest for the MLP. Not leaving out any metabolites from input spectra resulted in mixed results for the MLP model across test examples, while performance generally improved for the CNN and transformer. Leaving out more metabolites had little effect on MLP performance, had mixed results on CNN performance, and improved transformer performance. Extending the concentration range resulted in slight accuracy improvement for the MLP and transformer model. In summation, the larger dataset, more metabolites left out, and extended range modifications were considered the most promising modifications, especially given their performance for the mimic tissue range example that most closely resembles our target metabolomics task. Moving forward, we refer to an “improved combined distribution” dataset that employs this combination of modifications (although only 20,000 training/validation spectra are used in the loss function and hyperparameter optimization phases to facilitate faster model training before switching to an even larger dataset of 250,000 for a final model comparison).

### 3.2. Parameter Optimization Phase

Loss functions were the next parameter varied to examine their effects on NN model performance in analyte quantification in NMR spectra. Many loss functions were used to train MLP, CNN, and transformer networks on 20,000 spectra generated in the manner of the improved combined distribution dataset, and MAPE was determined on 10 test spectra from the uniform, low concentration, and mimic tissue range distributions, respectively. Mean MAPE and confidence intervals are plotted in Supplementary Figure S9 (for all losses except MSLE, which was left out of the figure due to very high error), and mean MAPE values are displayed in Supplementary Table S4. The MAPE loss function was very poor across all three models on all test spectra, and MSLE loss was extremely poor for the MLP and CNN models and moderately poor for the transformer. Most loss functions performed reasonably well on the uniformly distributed test spectra, but many saw performance drop on the low concentration and mimic tissue range test spectra. For the MLP and transformer, RAE and quantile loss with a q-value of 0.5 were consistently the most accurate on the low concentration and mimic tissue range spectra, while for the CNN, only quantile loss with a q-value of 0.5 stood out as the most accurate option on the mimic tissue range test spectra. Thus, RAE was selected for the MLP and transformer moving forward, and quantile loss with q = 0.5 was used for the CNN.

The MLP, CNN, and transformer models were next subjected to Bayesian hyperparameter optimization to fine-tune model architectures and training parameters using the improved combined distribution training dataset of 20,000 spectra and the loss functions determined in the prior experiment. The parameters determined using Optuna are displayed in Table 2 (the exact parameters used in the 100 trials are included as tables in the Supplementary Files).

**Figure 5:**
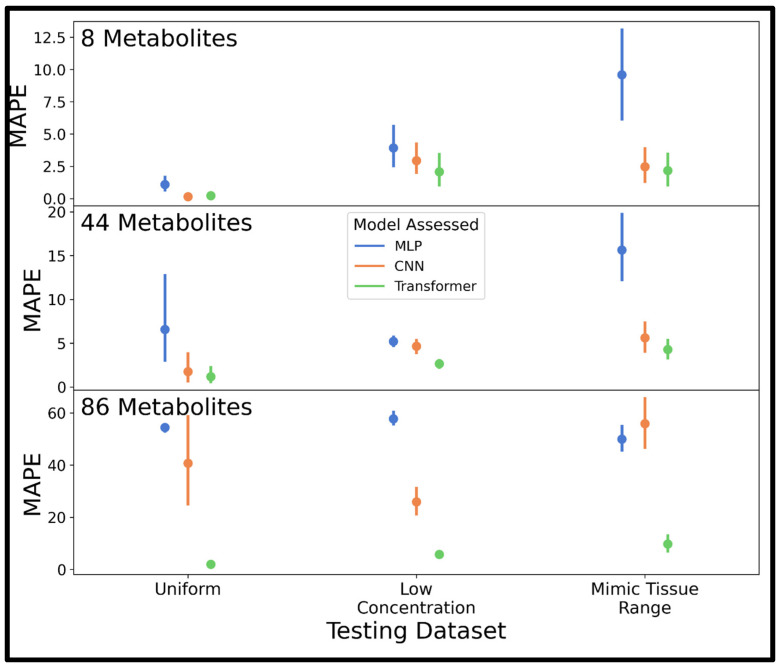
Pointplots displaying the mean and 95% confidence interval of the MAPE determined for the MLP, CNN, and transformer models trained using the improved combined distribution dataset of 250,000 spectra with Bayesian optimized hyperparameters. MAPE was determined on 10 test spectra generated using the uniform, low concentration, and mimic tissue range distributions. Abbreviations: MAPE = mean absolute percent error; MLP = multi-layered perceptron; CNN = convolutional neural network.

These sets of parameters were used to train an MLP, CNN, and transformer for quantification of metabolites in spectra of varying complexity (8, 44, or 86 analytes) using the improved combined distribution dataset with 250,000 spectra, and MAPE was determined on 10 testing spectra from the uniform, low concentration, and mimic tissue range distributions, respectively. Mean MAPE values and confidence intervals are displayed in Figure 5, where the transformer model outperforms the other models on all three test sets, followed in performance by the CNN (mean MAPE values are also shown in Table S5). The transformer especially outperformed the other models in highly complex spectra of 86 metabolites, and the MLP underperformed on test spectra with a high dynamic range of analyte concentrations as in the mimic tissue range examples. Hence, only the transformer model was utilized in further experiments.

### 3.3. Model Testing for Low- and High-Field Simulated Spectra

**Figure 6:**
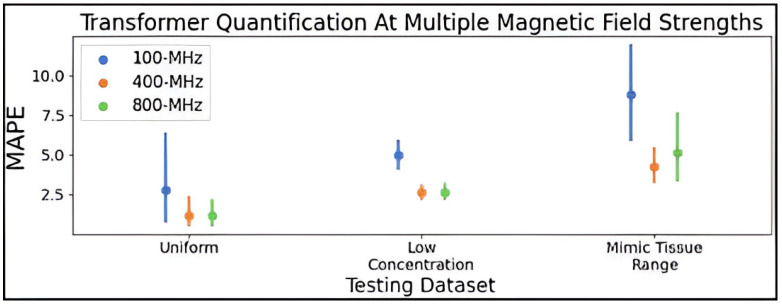
Point plot displaying the mean and 95% confidence interval of the MAPE determined for the hyperparameter optimized transformer trained using the improved combined distribution dataset of 250,000 simulated spectra at 100, 400, and 800-MHz. MAPE was determined on 10 test spectra generated using the uniform, low concentration, and mimic tissue range distributions. Abbreviations: MAPE = mean absolute percent error.

The hyperparameter-optimized transformer was next trained for metabolite profiling using 250,000 simulated mixture spectra at 100- and 800-MHz. Figure 6 compares the MAPE means with confidence intervals for the transformer at 100-, 400-, and 800-MHz, and it is shown that the 400-MHz model achieves higher accuracy among the three models, followed by the 800-MHz model. The mean MAPE values are listed in Supplementary Table S6.

## 4. Discussion

This research explored dataset considerations and parameter optimization for MLP, CNN, and transformer NNs for the task of quantifying analytes in 400-MHz 1D ^1^H-NMR spectra of metabolite mixtures. All three models were trained with various training dataset concentration distributions and dataset modifications before optimizing hyperparameters and finally deciding which model achieved superior performance. The best model was further assessed for quantification of metabolites at 100- and 800-MHz.

The concentration distribution phase of dataset exploration revealed that a combination of concentration distributions was effective in making all three NN models robust to various test spectra scenarios. This effect was strongest for the CNN, which performed optimally on all examples when using the combined distribution. The MLP and transformer both achieved high performance when trained using the mimic tissue range, high dynamic range, and combined distributions, especially the transformer model demonstrating the best performance overall. The combined distribution was selected moving forward given it was a consistently accurate method among the three models; although, for the MLP and transformer, the mimic tissue range and high dynamic range distributions should not be considered unpromising in future studies analyzing complex spectra with both very high and very low intensities. All three models were fast enough for real-time quantification of metabolites in NMR spectra, with each capable of profiling metabolites in 5000 spectra in milliseconds.

The dataset modification phase of training revealed several dataset alterations that improved analyte quantification accuracy for all or several NNs used in this study. As expected, increasing the dataset size improved performance for all models, especially the CNN and transformer, which conventionally perform well with very large datasets. The MLP saw mixed, non-conclusive results in the testing datasets after training on data modified to not leave out any metabolites or to leave out even more metabolites, while the CNN improved when no metabolites were left out, but the results were not significantly improved when leaving out more metabolites. The transformer improved when both leaving out more or not leaving out metabolites, a trend that may be because the test sets contain all 44 metabolites in all spectra to facilitate MAPE computation. Thus, training on all 44 metabolites every time is more representative of the test set, while training on the dataset where more metabolites are left out might promote model understanding of each metabolite’s signature chemical shifts and line shapes, given the increased potential for an absence of overlap. Extending the upper limit of the concentration range seen in training improved transformer performance without significantly impacting MLP and CNN accuracy. The log transformation was detrimental to the performance of all three models on all three testing datasets. In summary, the dataset modification experiments showed modifications like increasing dataset size, leaving out more metabolites, and extending the concentration range past the expected use range could improve NN performance.

Ten loss functions were assessed for model training in the task of analyte quantification, focusing on a variety of losses with different sensitivities to outliers and the scale of target values given the large range and expected distribution of concentrations generally encountered in NMR-based metabolomics studies. Results confirmed that RAE achieved the highest accuracy for the MLP and transformer, while quantile loss with q = 0.5 was effective for the CNN and transformer.

The final phase of NN development for all three models was hyperparameter optimization. Bayesian hyperparameter optimization results guided the model architectures and training parameters used to train an MLP, CNN, and transformer using a larger dataset than previously (250,000 compared to 20,000 or 50,000 used prior) for a final comparison to decide the most accurate model in spectra of varying complexity (8, 44, or 86 metabolites). Consistent with previous experiments in the current study, the transformer was the highest-performing model across all three testing sets regardless of spectral complexity, although the CNN was competitive at 8 and 44 analytes. These results agree with a trend observed in many applications where transformers are surpassing the benchmarks set by other NN architectures [42,43].

The hyperparameter-optimized transformer was additionally validated on spectra simulated at 100-MHz, relevant to benchtop NMR spectroscopy, and 800-MHz, relevant to high-resolution NMR spectroscopy. The transformer saw a modest decrease in performance in quantifying analytes in 100-MHz spectra compared to 400-MHz, while the model trained for quantification at 800-MHz was comparable to the network trained on 400-MHz data. This drop in accuracy at 100-MHz is likely due to the lower SNR and lower spectral resolution in low-field spectra where coupling constants increase beyond peak dispersion relative to the 400- and 800-MHz spectra. It is a promising result that the transformer could quantify 86 metabolites at 100-MHz with only a minor drop in accuracy compared to 800-MHz. It is possible that optimizing hyperparameters on 100- and 800-MHz spectra might improve performance beyond what was achieved using the parameters selected using 400-MHz spectra.

The results of this study emphasize important aspects of applying NNs for NMR metabolite profiling. The comparison of training dataset concentration distributions confirmed that training using samples drawn from multiple distributions as opposed to a single distribution leads to models that are more robust to varied input distributions. The dataset modification phase of model development confirmed that the proportion of analytes left out of samples in the training dataset affected model performance and that extending the concentration range of training data beyond the expected concentration range of inputs can improve quantitative performance. The comparison of optimized models, consistent with results throughout the study, revealed the transformer to be more effective than the MLP or CNN for analyte quantification in NMR spectra, especially as the complexity of spectra increases due to a larger number of metabolites. Finally, results show that the transformer approach can be effective at multiple field strengths, even at field strengths compatible with benchtop NMR.

This work presents a general workflow that could be used to train models for the quantification of NMR spectra, but this work also has important limitations. Despite efforts to promote realism in spectra, including residual protein/lipid signals and more realistic chemical shift dependencies (such as shifting metabolites/peaks based on temperature, pH, concentration, and metabolite interactions) would be invaluable to improving the approach. Improving chemical shift dependencies through NMR simulation methods, empirical rules applied during data augmentation, or otherwise is a critical next step if NNs like those designed in this study are to be applied to experimentally acquired NMR spectra. Using simulated spectra is a limitation compared to proving the method in experimentally acquired spectra; however, the simplicity and customizability afforded by simulated spectra are preferable for an extensive investigation of dataset and model development and validation as was performed in this study. The primary limitation of experimental data is that one cannot easily acquire enough spectra to train a model (tens of thousands to millions of correctly acquired spectra containing accurately weighted compounds). It is possible to obtain experimental spectra of metabolite reference standards individually and use these to generate data as performed in this study with simulated spectra. This would not overcome chemical shift dependencies as described above but could work for some well-defined systems, such as for NMR lipid profiling, as demonstrated in a previous study applying NNs to an experimentally acquired NMR metabolomics dataset [7]. An alternative approach that could help overcome chemical shift dependency limitations could be to train a model using synthetic spectra, using either a similar generation method as used in this study, i.e., using experimental spectra of individual standards as in Johnson et al., which was successful in quantifying lipids in experimentally acquired spectra of hepatic tissue extracts [7], or likely the best case would be using simulated spectra as in this study but with more accurate chemical shift dependencies incorporated and then to fine-tune the model on tens to hundreds of experimental spectra. In this way, one could train entirely on synthetic spectra and then adjust model parameters to achieve better performance on experimentally acquired spectra, which already manifest signal effects from pH, concentration, metabolite interaction effects, etc. Fine-tuning may even be a way to tailor quantification to a given scenario such as optimizing quantification for a single NMR instrument or a specific media.

To confirm the best practices determined in this manuscript, these methods should be validated in experimentally acquired metabolomics data. Further, benchmarking the performance against popular peak-fitting methods such as Chenomx is an important next step to confirming if NN methods are superior. To further improve applicability, it should be validated whether a NN such as a transformer could learn hundreds of potential metabolites and still maintain accuracy in examples comparable to those seen in this study (e.g., can a model learn 500 metabolites and maintain accuracy in realistic mixtures of ~30–80 metabolites).

Beyond the methods assessed in this work, further model architectures or modifications are worth consideration, such as encoder/decoder transformers, convolutional transformers, CRNNs, or ensembles of models (for averaging numerous models for predictions, or potentially to ensemble models aimed at different concentration ranges), and dataset modifications such as alternative concentration distributions, time/domain input data, and datasets with 1 million or more training spectra. With NNs, it is also possible to design a model that takes in multidimensional NMR spectra or a NN that takes in multiple spectra for a single metabolite quantification task (i.e., input 1D ^1^H, ^13^C, and ^31^P spectra to obtain a single metabolite profile for given sample). The attention mechanism could potentially be modified for improved performance, perhaps having all data points attend one another (computationally expensive) or some sort of learned or custom attention where all resonances of an analyte attend one another and potentially some overlapping signals among other data points. The attention mechanism could potentially be replaced by another mechanism, such as the recently introduced Hyena operators [44]. Other model improvements might include using explainable artificial intelligence techniques to discern what input features were most important for analyte quantification [45], or in the case of transformers, attention maps could serve a similar purpose [46]. Adding a peak alignment step prior to input into the NN could potentially improve model accuracy and alignment could be a method to limit the detrimental effects of not incorporating chemical shift effects of temperature, pH, metabolite interactions, etc., in training data. The methods from this manuscript could alternatively be applied to other quantitative NMR spectroscopic analyses such as molecular weight determination [27], or potentially to other forms of spectroscopy.

## 5. Conclusions

This study concludes that the transformer was the most effective NN for NMR metabolite quantification, especially as the number of metabolites per spectra increased or target concentrations were low or had a large dynamic range, and the transformer was able to accurately quantify metabolites in simulated spectra from 100-MHz up to 800-MHz. By carefully considering how the dataset and model are constructed, models like CNNs and especially transformers achieve highly accurate metabolite profiling in very complex spectra with a large dynamic range of concentrations. The methods developed in this manuscript make a strong case that NNs, especially transformers, are an excellent choice for the next generation of automated, quantitative NMR-based metabolomics software.

## Figures and Tables

**Table 1:** Varied parameters and their respective search ranges utilized in Bayesian hyperparameter optimization for the MLP, CNN, and transformer models in the task of analyte quantification in NMR spectra.

MLP		CNN		Transformer	
Parameter	Search Range	Parameter	Search Range	Parameter	Search Range
**# of Hidden Layers**	1–4	**# of Conv.** **Layers**	1–4	**Binning Size**	100/200/500/ 1000/2000
**Activation** **Function**	ReLU/ LeakyReLU/eLU	**Conv. Kernel** **Size**	3–12	**Embedding** **Dimension Size**	256/512/1024
**Hid. Layer 1 –** **# of Nodes**	10–1000	**Conv. Kernel** **Stride**	1–8	**# of Attention** **Heads**	1/2/4/8/16
**Hid. Layer 2 –** **# of Nodes**	10–1000	**Channels per** **Conv. Layer**	5–50	**# of Encoding** **Layers**	1–9
**Hid. Layer 3 –** **# of Nodes**	10–1000	**Pooling Type**	None/Max/ Average	**# of Feedforward** **Layers**	1–2
**Hid. Layer 4 –** **# of Nodes**	10–1000	**Pooling Stride**	1–2	**Feedforward Size**	512/1024/2048
**Learning Rate**	1 × 10^−5^–1 × 10^−1^	**Feedforward Size**	100–250	**Learning Rate**	1 × 10^−6^–1 × 10^−2^
**Regularization** **Strength**	1 × 10^−6^–1 × 10^−2^	**Learning Rate**	1 × 10^−6^–1 × 10^−2^	**Regularization** **Strength**	1 × 10^−6^–1 × 10^−2^
**Batch Size** **(Effective)**	64–1024	**Regularization** **Strength**	1 × 10^−6^–1 × 10^−2^	**Batch Size** **(Effective)**	32–512
**-**	-	**Batch Size** **(Effective)**	64–512	**-**	-

Abbreviations: # = number, Hid. = hidden, Conv. = convolutional, MLP = multi-layered perceptron, and CNN = convolutional neural network.

**Table 2:** Optimal MLP, CNN, and transformer model parameters determined with Bayesian optimized hyperparameters.

MLP		CNN		Transformer	
Parameter	Optimal Parameter	Parameter	Optimal Parameter	Parameter	Optimal Parameter
**# of Hidden Layers**	2	**# of Conv.** **Layers**	3	**Binning Size**	500
**Activation** **Function**	LeakyReLU	**Conv. Kernel** **Size**	10	**Embedding** **Dimension Size**	512
**Hid. Layer 1 –** **# of Nodes**	222	**Conv. Kernel** **Stride**	4	**# of Attention** **Heads**	16
**Hid. Layer 2 –** **# of Nodes**	463	**Channels per** **Conv. Layer**	35	**# of Encoding** **Layers**	1
**Hid. Layer 3 –** **# of Nodes**	-	**Pooling Type**	None	**# of Feedforward** **Layers**	1
**Hid. Layer 4 –** **# of Nodes**	-	**Pooling Stride**	-	**Feedforward Size**	512
**Learning Rate**	2.1 × 10^−3^	**Feedforward Size**	233	**Learning Rate**	5.0 × 10^−4^
**Regularization** **Strength**	9.4 × 10^−3^	**Learning Rate**	2.1 × 10^−5^	**Regularization** **Strength**	9.6 × 10^−3^
**Batch Size** **(Effective)**	124	**Regularization** **Strength**	5.0 × 10^−3^	**Batch Size** **(Effective)**	244
**-**	-	**Batch Size** **(Effective)**	64	**-**	-

Abbreviations: # = number; Conv. = convolutional; MLP = multi-layered perceptron; CNN = convolutional neural network.

## Data Availability

The simulated spectra and code used in this study are available on GitHub (https://github.com/tpirneni/DL-NMR, accessed 10 January 2025).

## Supplementary Materials

The following supporting information can be downloaded at: https://www.mdpi.com/article/10.3390/metabo15040249/s1. Figure S1: Forty-four metabolite signals individually and as a synthetic mixture spectrum; Figure S2: Experimental and simulated spectra SNR comparison; Figure S3: Zoomed 100-MHz spectra of an NADP and nicotinic mononucleotide signal with various data augmentations applied; Figure S4: Zoomed 400-MHz spectra of an NADP and nicotinic mononucleotide signal with various data augmentations applied; Figure S5: Zoomed 800-MHz spectra of an NADP and nicotinic mononucleotide signal with various data augmentations applied; Figure S6: 100-MHz spectra of a synthetic 44 metabolite mixture with various data augmentations applied; Figure S7: 400-MHz spectra of a synthetic 44 metabolite mixture with various data augmentations applied; Figure S8: 800-MHz spectra of a synthetic 44 metabolite mixture with various data augmentations applied; Figure S9: Mean MAPE determined using the MLP, CNN, and transformer model trained using ten different loss functions; Figure S10: Example of a mixture spectrum before and after log-transformation; Table S1: List of simulated metabolite signals downloads from HMDB for generating synthetic NMR spectra; Table S2. Mean MAPE for MLP, CNN, and transformer model trained with the various concentration distributions; Table S3. Mean normalized MAPE for MLP, CNN, and transformer model trained with the various dataset modifications; Table S4: Mean MAPE determined using the MLP, CNN, and transformer model trained using ten different loss functions; Table S5: Mean MAPE values determined using the hyperparameter-optimized MLP, CNN, and transformer model trained with 250,000 spectra for quantification of up to 8, 44, and 86 metabolites. Table S6: Mean MAPE values determined using the transformer model trained with 250,000 spectra generated at 100-, 400-, or 800-MHz.
