# Supplementary material for: Towards Optimizing Neural Network-Based Quantification for NMR Metabolomics
Source: Metabolites. 2025 Apr 4;15(4):249. doi: 10.3390/metabo15040249 (PMC12029129; doi:10.3390/metabo15040249)
Supplement: Supplementary file 1 [file metabolites-15-00249-s001.zip › metabolites-3478457-supplementary.pdf]

Supplementary Materials

Towards Optimizing Neural Network-Based Quantification for NMR Metabolomics

Hayden Johnson [1] and Aaryani Tipirneni-Sajja [1,2,*]

[1] Department of Biomedical Engineering, The University of Memphis, Memphis, TN 38152, USA; htjhnson@memphis.edu
[2] Department of Biomedical Engineering, University of Houston, Houston, TX 77004, USA
* Correspondence: asajja@uh.edu

Table S1. Metabolites selected for generating synthetic NMR spectra from simulated proton NMR spectra downloaded from the HMDB.

8 Metabolites	44 Metabolites	86 Metabolites
choline	choline	choline
glutathione	glutathione	glutathione
itaconic acid	itaconic acid	itaconic acid
nicotinic acid mononucleotide	nicotinic acid mononucleotide	nicotinic acid mononucleotide
phosphocreatine	phosphocreatine	phosphocreatine
methionine	methionine	methionine
creatine	creatine	creatine
L-asparagine	L-asparagine	L-asparagine
	alpha-D-glucose	alpha-D-glucose
	beta-D-glucose	beta-D-glucose
	acetic acid	acetic acid
	glycine	glycine
	L-alanine	L-alanine
	lysine	lysine
	inosine	inosine
	pyruvic acid	pyruvic acid
	creatinine	creatinine
	leucine	leucine
	nicotinamide adenine dinucleotide (NAD)	nicotinamide adenine dinucleotide (NAD)
	niacinamide	niacinamide
	3-hydroxybutryic acid	3-hydroxybutryic acid
	acetoacetic acid	acetoacetic acid
	citric acid	citric acid
	fructose-6-phosphate	fructose-6-phosphate
	glutamic acid	glutamic acid
	L-tyrosine	L-tyrosine
	proline	proline
	L-threonine	L-threonine
	isoleucine	isoleucine
	serine	serine
	L-aspartic acid	L-aspartic acid
	isocitric acid	isocitric acid
	oxoglutaric acid	oxoglutaric acid
	nicotinamide adenine dinucleotide (NADP)	nicotinamide adenine dinucleotide (NADP)
	oxalacetic acid	oxalacetic acid
	taurine	taurine
	L-arginine	L-arginine
	glutamine	glutamine
	L-tryptophan	L-tryptophan
	succinyl-CoA	succinyl-CoA
	fructose 1,6-bisphosphate	fructose 1,6-bisphosphate
	flavin adenine dinucleotide with hydrogen (FADH)	flavin adenine dinucleotide with hydrogen (FADH)
	acetyl-CoA	acetyl-CoA

	flavin adenine dinucleotide (FAD)	flavin adenine dinucleotide (FAD)
	reduced nicotinamide adenine dinucleotide (NADH)	reduced nicotinamide adenine dinucleotide (NADH)
		2-Hydroxybutyric acid
		alpha-ketoisovaleric acid
		adenine
		adenosine monophosphate
		dimethylglycine
		ethanol
		glycerol
		fumaric acid
		phenylalanine
		D-mannose
		inosinic acid
		L-acetylcarnitine
		myo-inositol
		ornithine
		sarcosine
		uridine 5'-monophosphate
		uridine
		3-methyl-2-oxovaleric acid
		acetylglycine
		adenosine triphosphate
		L-valine
		lactic acid
		maleic acid
		xanthine
		hypoxanthine
		formic acid
		1-methylhistidine
		succinic acid
		L-cysteine
		D-alpha-aminobutyric acid
		ketoleucine
		isovaleric acid
		3-hydroxyisovaleric acid
		trimethylamine N-oxide
		ADP
		acetone
		caffeine
		methanol
		histidine
		propylene glycol
		selenocysteine
		oxidized glutathione

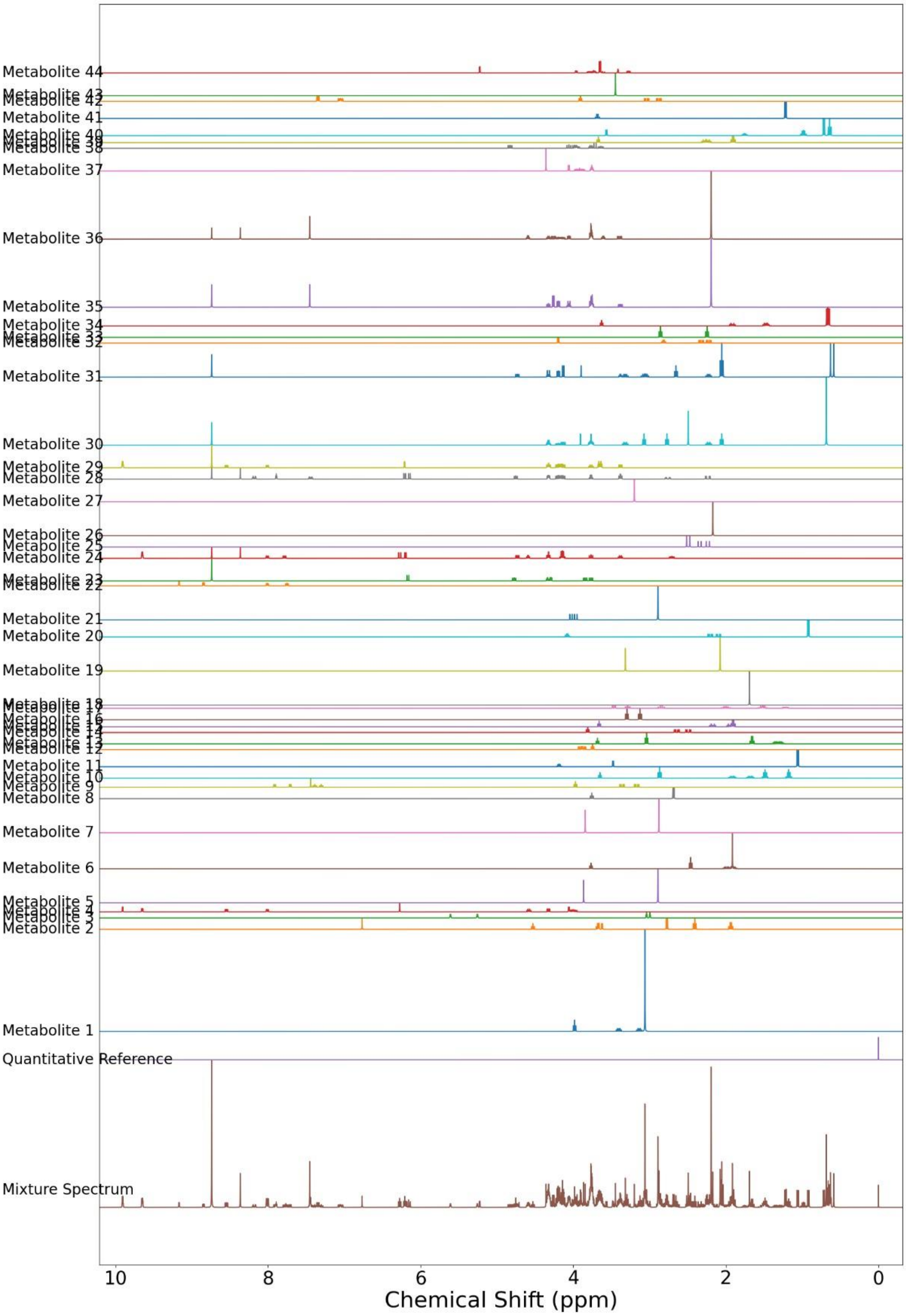

Figure S1. Simulated metabolite proton NMR spectra as downloaded from the HMDB (first 44 spectra), a quantitative reference spectrum mimicking TSP-d4, and a mixture spectrum of all 44 metabolites. All metabolites are at equal concentrations in all spectra.

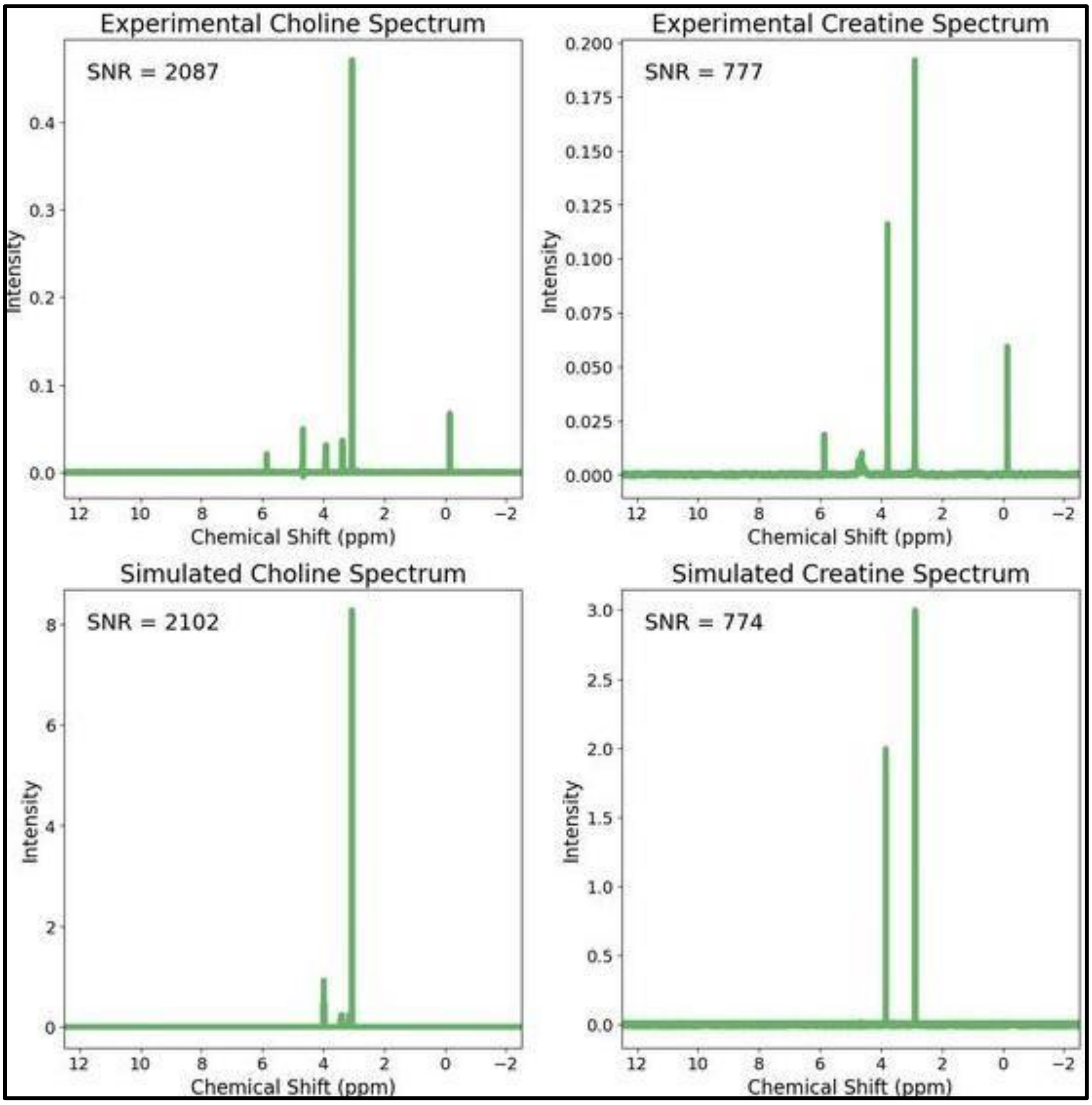

Figure S2. Experimentally acquired spectra of choline and creatine at 400-MHz (top panels) and simulated spectra of choline and creatine at 400-MHz (bottom panels). The SNR was measured for the experimental spectra, and this SNR was used to determine how the noise magnitude to add to simulated spectra (for which SNR is also reported).

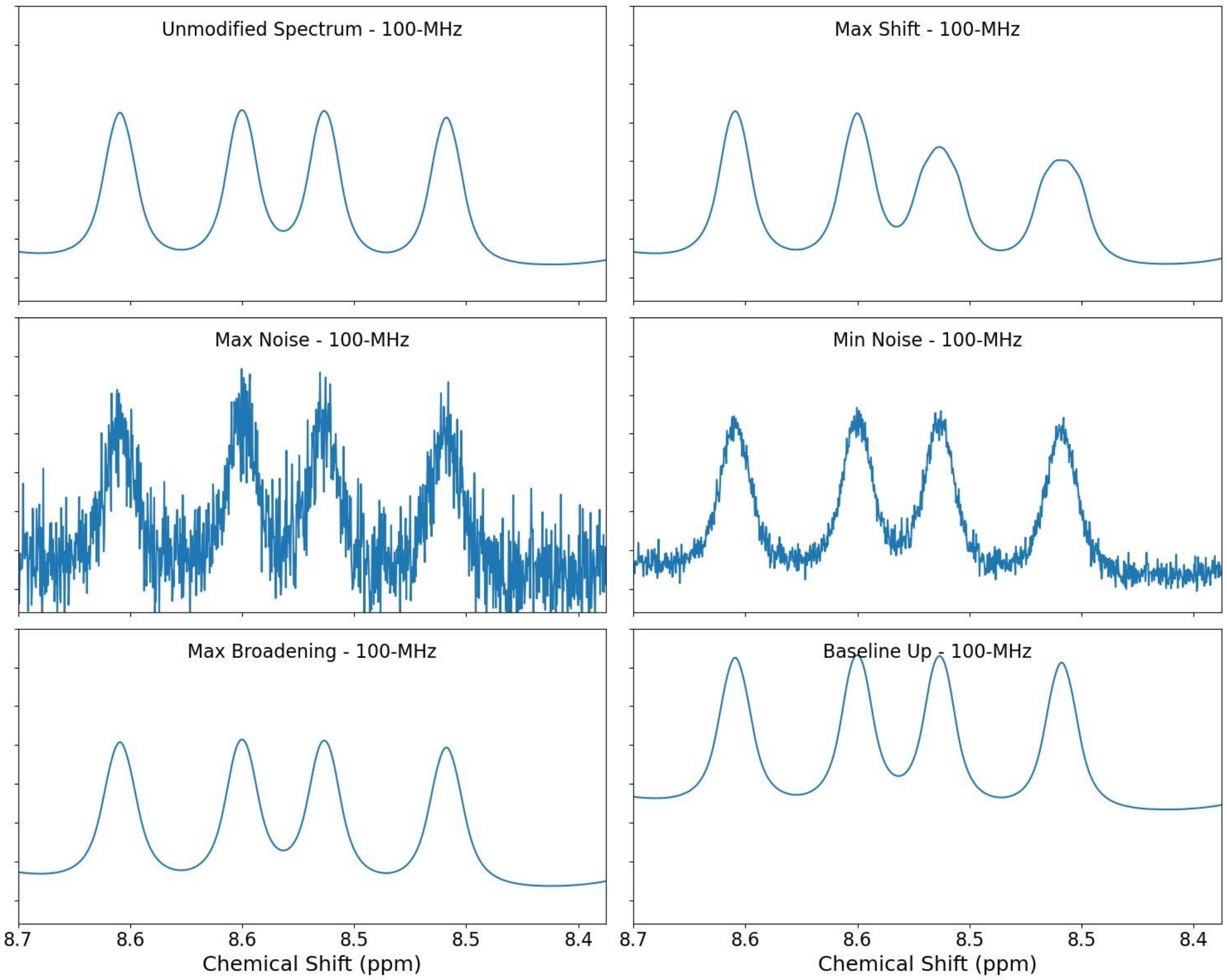

Figure S3. Zoomed in synthetic 100-MHz spectra for overlapped simulated NADP and nicotinic acid mononucleotide resonances at 1 mM before and after applying many of the augmentations utilized in generating synthetic spectra for neural network development. Displayed are unmodified peaks, the maximum shifting of peaks along the chemical shift axis, the maximum amount of noise applied, the minimum amount of noise applied, the maximum amount of line-broadening applied, and the maximum amount of baseline shift applied.

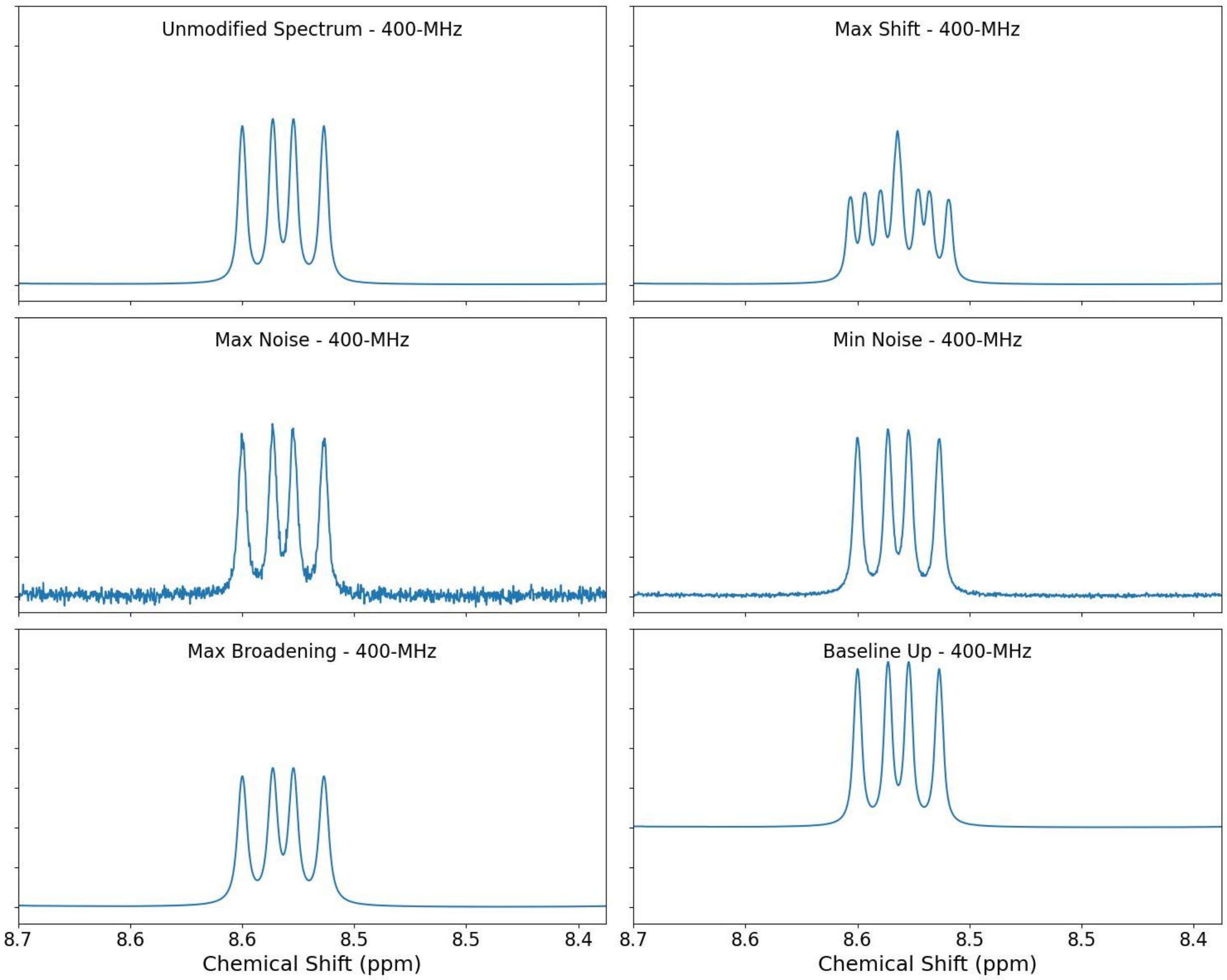

Figure S4. Zoomed in synthetic 400-MHz spectra for overlapped simulated NADP and nicotinic acid mononucleotide resonances at 1 mM before and after applying many of the augmentations utilized in generating synthetic spectra for neural network development. Displayed are unmodified peaks, the maximum shifting of peaks along the chemical shift axis, the maximum amount of noise applied, the minimum amount of noise applied, the maximum amount of line-broadening applied, and the maximum amount of baseline shift applied.

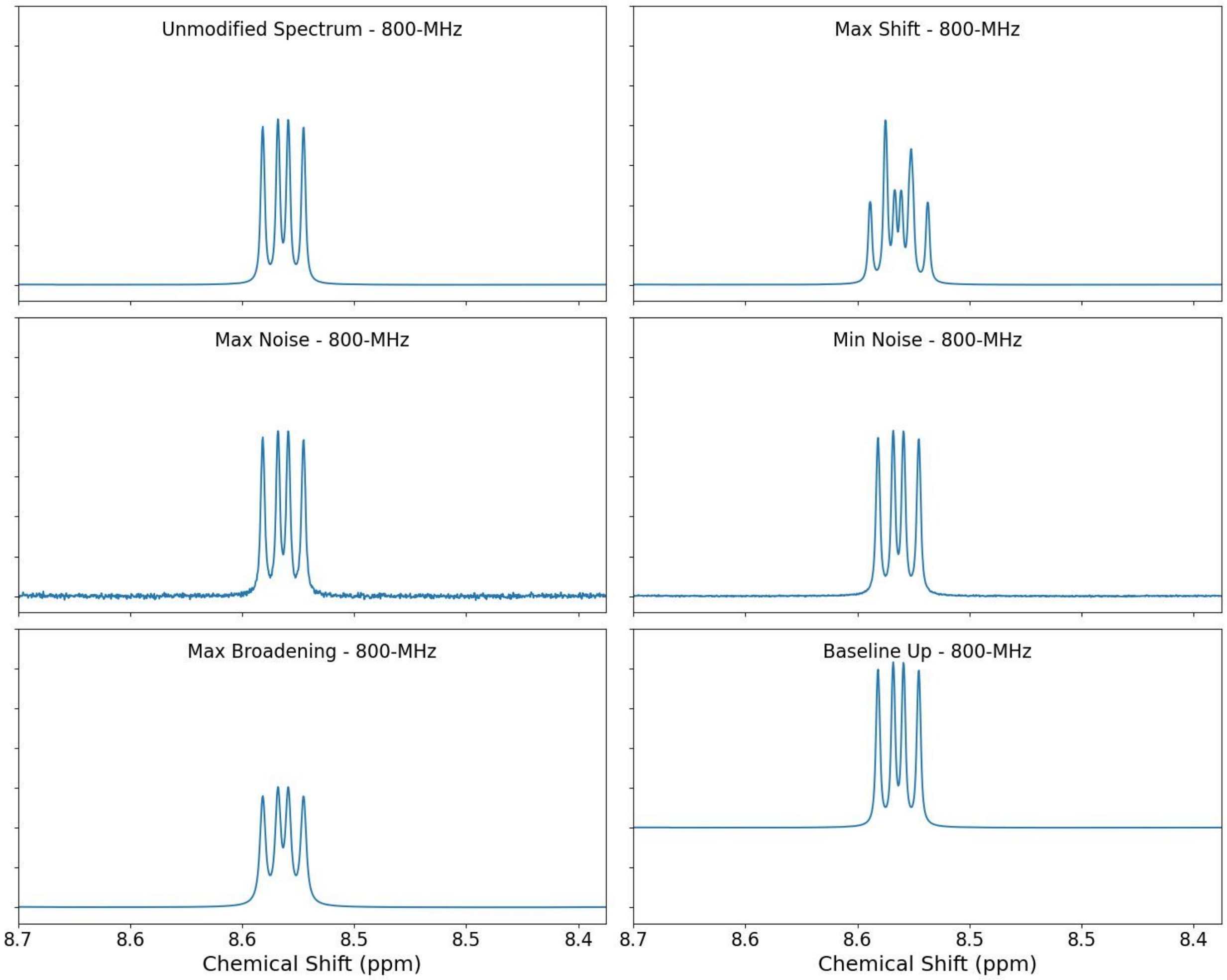

Figure S5. Zoomed in synthetic 800-MHz spectra for overlapped simulated NADP and nicotinic acid mononucleotide resonances at 1 mM before and after applying many of the augmentations utilized in generating synthetic spectra for neural network development. Displayed are unmodified peaks, the maximum shifting of peaks along the chemical shift axis, the maximum amount of noise applied, the minimum amount of noise applied, the maximum amount of line-broadening applied, and the maximum amount of baseline shift applied.

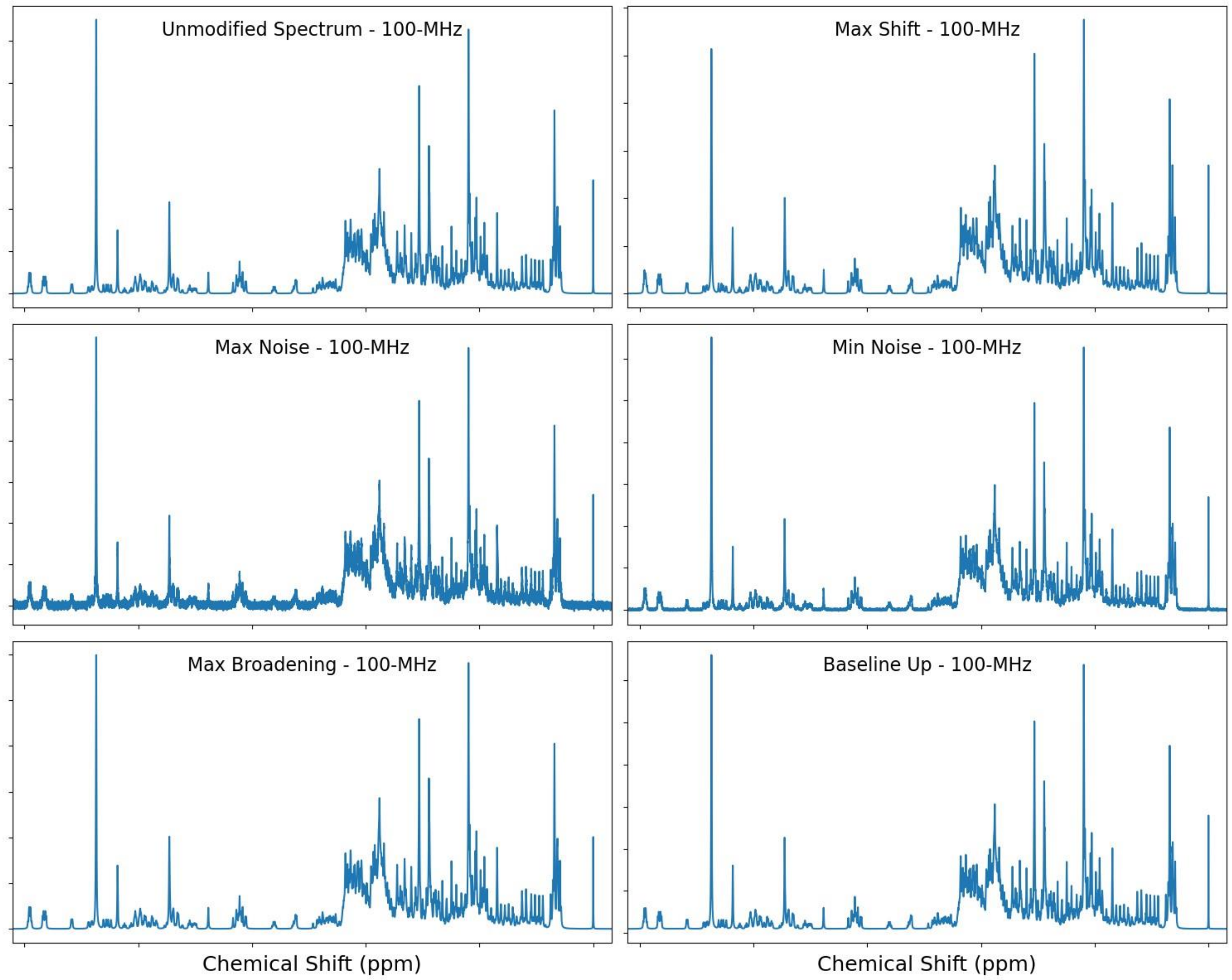

Figure S6. Synthetic 100-MHz spectra of 44 metabolites all at 1 mM before and after applying many of the augmentations utilized in generating synthetic spectra for neural network development. Displayed are unmodified peaks, the maximum shifting of peaks along the chemical shift axis, the maximum amount of noise applied, the minimum amount of noise applied, the maximum amount of line-broadening applied, and the maximum amount of baseline shift applied.

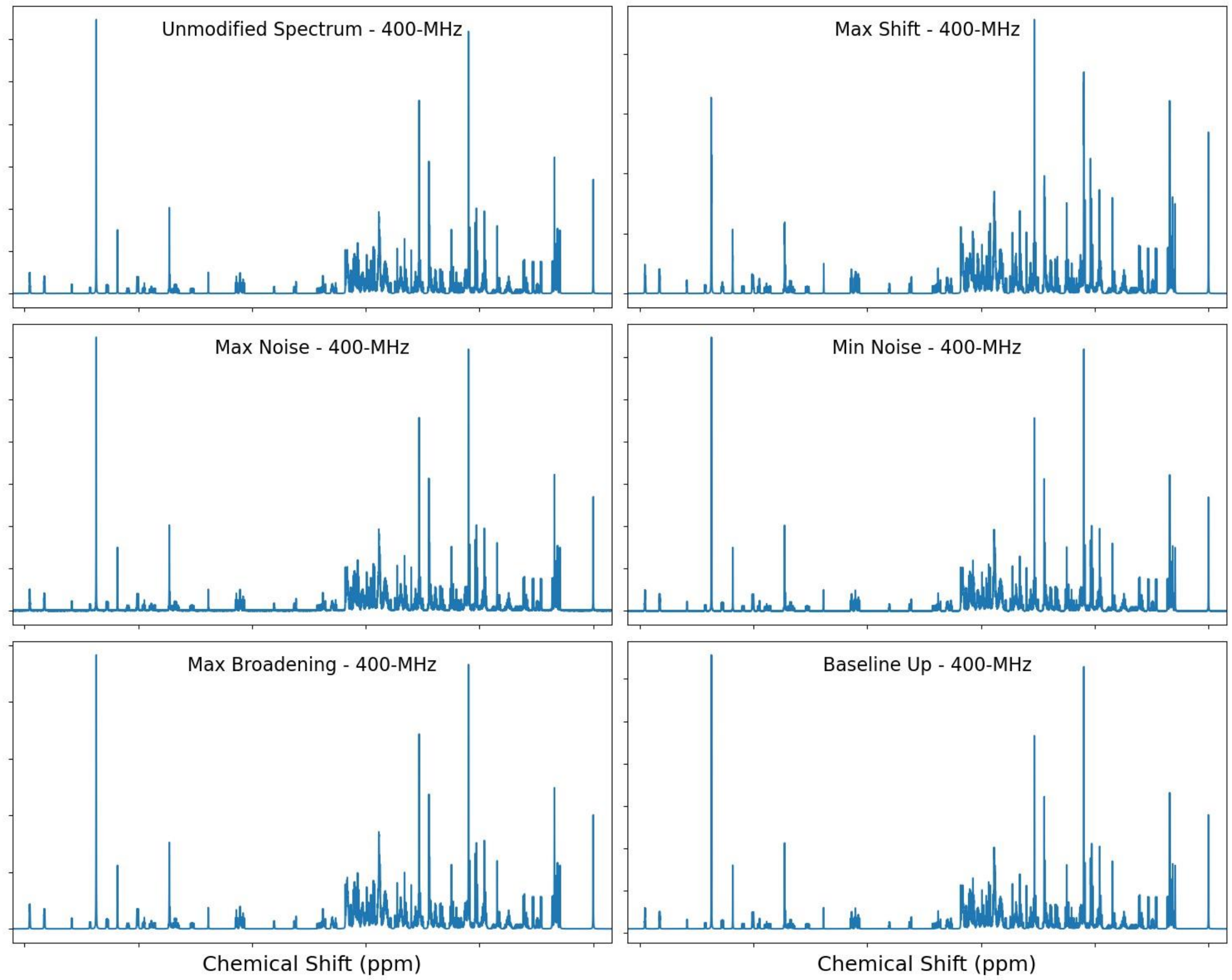

Figure S7. Synthetic 400-MHz spectra of 44 metabolites all at 1 mM before and after applying many of the augmentations utilized in generating synthetic spectra for neural network development. Displayed are unmodified peaks, the maximum shifting of peaks along the chemical shift axis, the maximum amount of noise applied, the minimum amount of noise applied, the maximum amount of line-broadening applied, and the maximum amount of baseline shift applied.

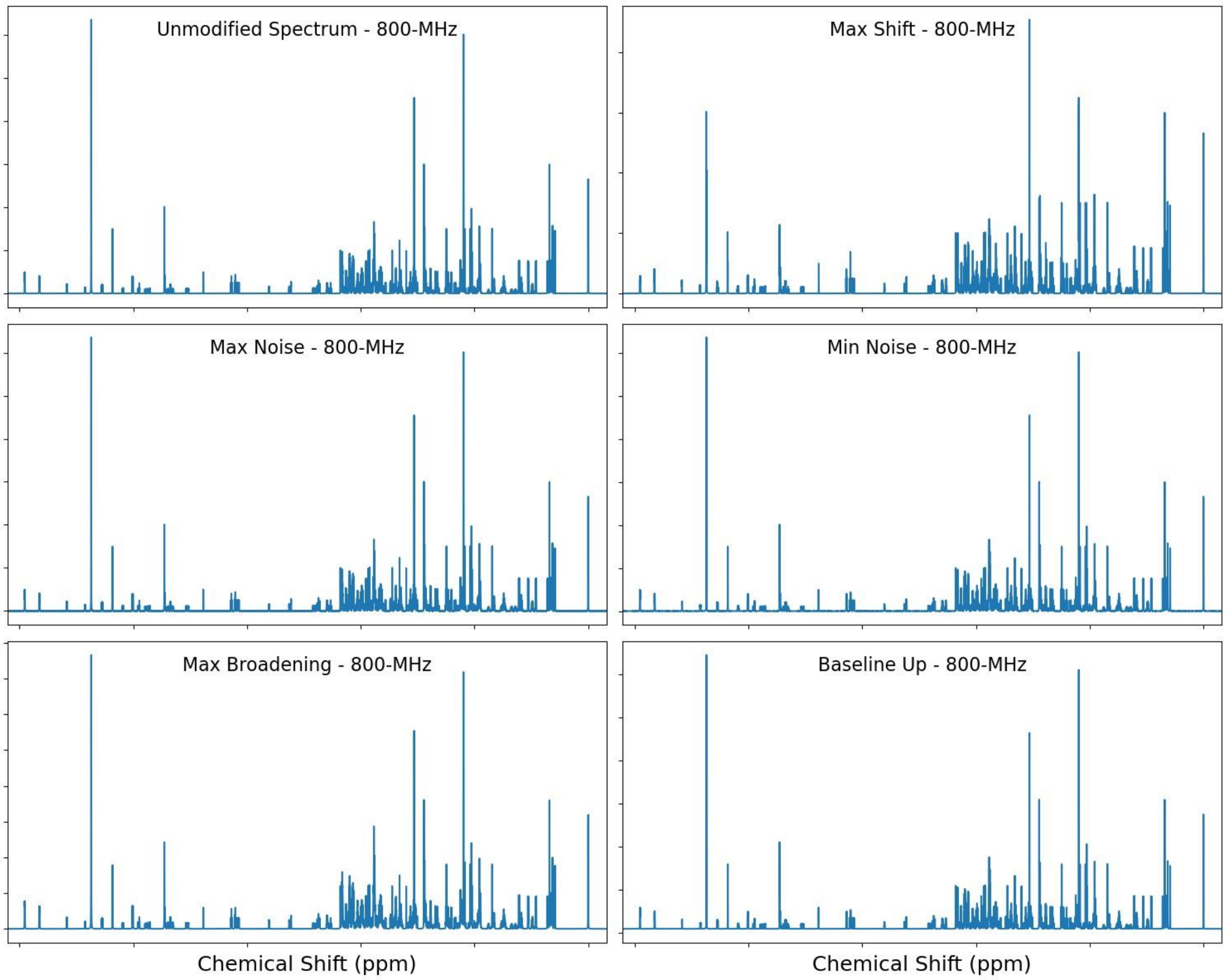

Figure S8. Synthetic 800-MHz spectra of 44 metabolites all at 1 mM before and after applying many of the augmentations utilized in generating synthetic spectra for neural network development. Displayed are unmodified peaks, the maximum shifting of peaks along the chemical shift axis, the maximum amount of noise applied, the minimum amount of noise applied, the maximum amount of line-broadening applied, and the maximum amount of baseline shift applied.

Table S2. Mean MAPE values determined using the MLP, CNN, and transformer model trained with either the uniform, mimic tissue range, high dynamic range, low concentration, or combined distribution datasets. Mean MAPE is determined on 10 test spectra generated using either a uniform, low concentration, or mimic tissue range concentration distribution. For each column (i.e., for each validation set), a color gradient from red to blue denotes high and low mean MAPE values, respectively.

		Uniform	Low Concentration	Mimic Tissue Range
Uniform	MLP	3.7	7.2	117.0
	CNN	3.3	40.7	41.9
	Transformer	2.3	50.8	28.1
Mimic Tissue Range	MLP	10.1	4.3	14.6
	CNN	22.7	20.9	39.1
	Transformer	15.6	4.5	10.1

High Dynamic Range	MLP	7.9	6.5	49.5
	CNN	30.1	36.0	58.4
	Transformer	2.3	6.2	8.3
Low Concentration	MLP	106.9	8.8	287.4
	CNN	100.8	62.9	118.4
	Transformer	98.9	5.2	96.2
Combined	MLP	7.8	6.2	24.2
	CNN	2.4	10.5	16.2
	Transformer	2.5	7.0	12.9

Abbreviations: MLP = multi-layered perceptron, and CNN = convolutional neural network; MAPE = mean absolute percent error.

Table S3. Mean normalized MAPE values determined using the MLP, CNN, and transformer model trained with the combined distribution dataset with one of the following modifications: log transformation, increasing dataset size, leaving no metabolites out, leaving more metabolites out, or extending the upper limit of the concentration range. Mean normalized MAPE is computed by dividing MAPE determined on 10 test spectra generated using either a uniform, low concentration, or mimic tissue range concentration distribution by the MAPE achieved prior to dataset modification.

		Uniform	Low Uniform	Mimic Tissue Range
Log Transformed Spectra	MLP	3.30	10.1	3.85
	CNN	1.28	1.08	1.01
	Transformer	1.30	1.16	1.26
Larger Dataset (50,000 spectra)	MLP	0.88	1.00	0.75
	CNN	0.83	0.81	0.58
	Transformer	0.62	0.70	0.61
No Metabolites Left Out	MLP	0.90	0.77	1.68
	CNN	0.99	0.69	0.63
	Transformer	0.71	0.80	1.02
More Metabolites Left Out	MLP	0.90	0.95	0.79
	CNN	1.62	0.95	0.87
	Transformer	0.77	0.66	0.75
Extended Concentration Range	MLP	0.97	0.83	0.97
	CNN	1.00	1.11	0.92
	Transformer	0.67	0.91	0.80

Abbreviations: MLP = multi-layered perceptron, and CNN = convolutional neural network; MAPE = mean absolute percent error.

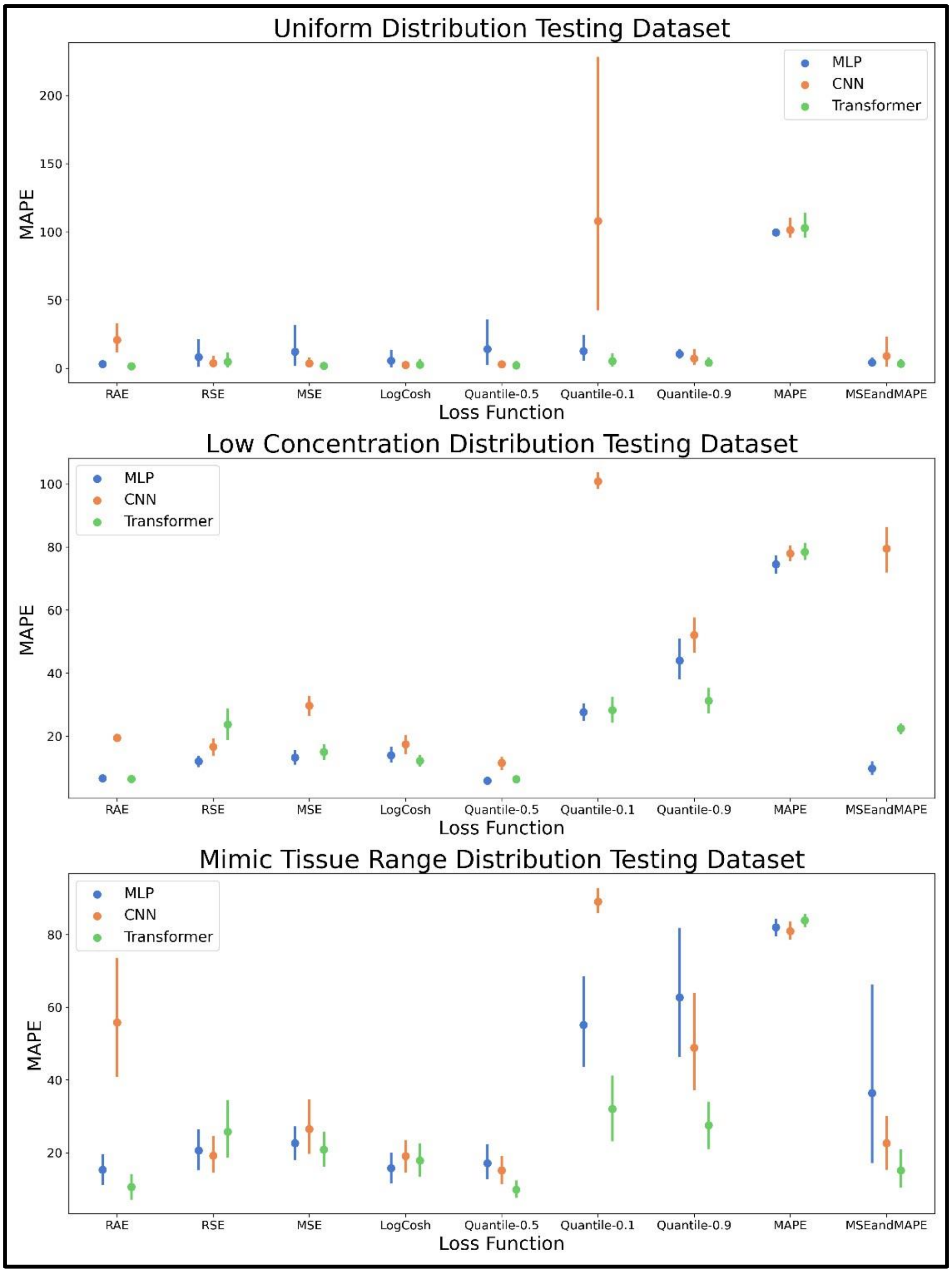

Figure S9. Mean MAPE values and confidence intervals determined using the MLP, CNN, and transformer model trained with the improved combined distribution dataset and one of the following loss functions: RAE, RSE, MSE, LogCosh, quantile (q = 0.1, 0.5, and 0.9), MAPE, and MSE/MAPE. When using MAPE and MAPE/MSE loss functions, no metabolites were left out of the training dataset to permit MAPE computation. Mean MAPE is determined on 10 test spectra generated using either a uniform, low concentration, or mimic tissue range concentration distribution. MSLE loss was also used, but was left off given its high MAPE values would increase the y-axis range make the plots less visually informative. Abbreviations: MLP = multi-layered perceptron, and CNN = convolutional neural network; MAPE = mean absolute percent error; RAE = relative absolute error; RSE = relative squared error; MSE = mean squared error; MSLE = mean squared logarithmic error.

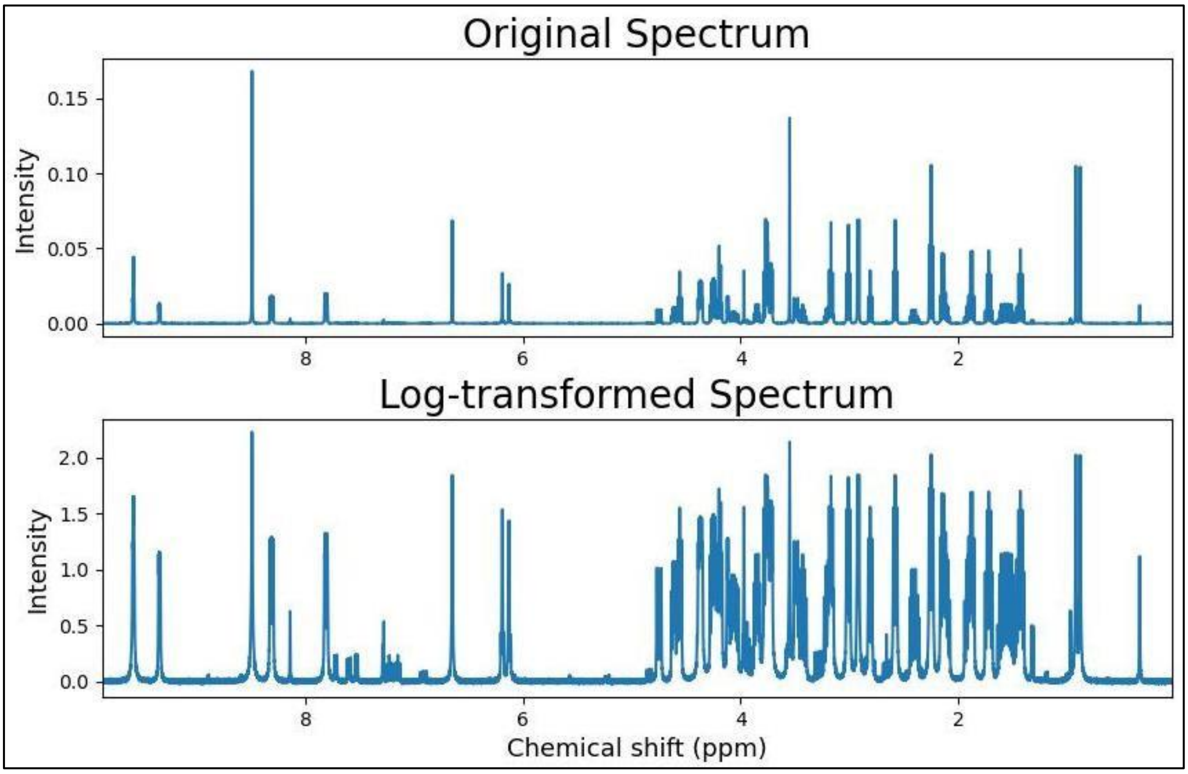

Figure S10. Example of a mixture spectrum before and after log-transformation.

Table S4. Mean MAPE values determined using the MLP, CNN, and transformer model trained with the improved combined distribution dataset and one of the following loss functions: RAE, RSE, MSE, LogCosh, quantile (q = 0.1, 0.5, and 0.9), MSLE, MAPE, and MSE/MAPE. When using MAPE and MAPE/MSE loss functions, no metabolites were left out of the training dataset to permit MAPE computation. Mean MAPE is determined on 10 test spectra generated using either a uniform, low concentration, or mimic tissue range concentration distribution. For each column (i.e., for each test set), a color gradient from red to blue denotes high and low mean MAPE values, respectively.

		Uniform	Low Uniform	Mimic Tissue Range
**RAE**	**MLP**	3.1	6.7	15.3
	**CNN**	20.7	19.5	55.8
	**Transformer**	1.5	6.5	10.6
**RSE**	**MLP**	8.2	12.0	20.6
	**CNN**	3.8	16.7	19.2
	**Transformer**	4.7	23.7	25.8
**MSE**	**MLP**	12.1	13.3	22.6
	**CNN**	3.6	29.7	26.5
	**Transformer**	1.8	15.0	20.8
**LogCosh**	**MLP**	5.5	13.9	15.8
	**CNN**	2.5	17.4	19.1
	**Transformer**	2.6	12.2	17.8
**Quantile-0.5**	**MLP**	14.1	5.9	17.1
	**CNN**	2.9	11.6	15.2
	**Transformer**	2.1	6.4	9.8
**Quantile-0.1**	**MLP**	12.6	27.6	55.1
	**CNN**	107.9	100.8	89.1
	**Transformer**	5.1	28.3	32.0
**Quantile-0.9**	**MLP**	10.4	44.0	62.7
	**CNN**	7.2	52.1	48.9
	**Transformer**	4.1	31.2	27.6
**MSLE**	**MLP**	97.9	161.9	360.7
	**CNN**	3304.4	341.4	496.1
	**Transformer**	7.2	14.3	34.2
**MAPE**	**MLP**	99.5	74.5	82.0
	**CNN**	101.3	78.0	81.0
	**Transformer**	102.9	78.4	83.9
**MSE/MAPE**	**MLP**	4.3	9.8	36.4
	**CNN**	9.0	79.5	22.6
	**Transformer**	3.3	22.5	15.1

Abbreviations: MLP = multi-layered perceptron, and CNN = convolutional neural network; MAPE = mean absolute percent error; RAE = relative absolute error; RSE = relative squared error; MSE = mean squared error; MSLE = mean squared logarithmic error.

Table S5. Mean MAPE values determined using the MLP, CNN, and transformer model trained with the hyperparameter optimized improved combined distribution dataset with 250,000 training/testing spectra. Results are shown for quantification using models trained with up to 8, 44, and 86 metabolites respectively. Mean MAPE is determined on 10 test spectra generated using either a uniform, low concentration, or mimic tissue range concentration distribution.

		**Uniform**	**Low Concentration**	**Mimic Tissue Range**
**8 Metabolites**	**MLP**	1.1	3.9	9.6
	**CNN**	0.15	3.0	2.5
	**Transformer**	0.23	2.1	2.2
**44 Metabolites**	**MLP**	6.6	5.2	15.6
	**CNN**	1.8	4.7	5.6
	**Transformer**	1.2	2.7	4.3
**86 Metabolites**	**MLP**	54.5	57.8	49.9
	**CNN**	40.1	25.9	55.9
	**Transformer**	2.0	5.6	9.8

Abbreviations: MLP = multi-layered perceptron, and CNN = convolutional neural network.

Table S6. Mean MAPE values (first three columns) determined using the transformer model trained with the hyperparameter optimized improved combined distribution dataset with 250,000 training/testing spectra generated at 100, 400, or 800-MHz. Mean MAPE is determined on 10 test spectra generated using either a uniform, low concentration, or mimic tissue range concentration distribution.

	Uniform	Low Uniform	Mimic Tissue Range
**100-MHz**	2.8	5.0	8.8
**400-MHz**	1.2	2.7	4.3
**800-MHz**	2.3	4.4	5.4
